# Life-Space in a National Cohort of U.S. Older Adults: Normative Data for the UAB Life-Space Assessment

**DOI:** 10.1093/geroni/igaa057.926

**Published:** 2020-12-16

**Authors:** Alexander Lo, Virginia Wadley, Michael Crowe, Cynthia Brown, Richard Kennedy

**Affiliations:** 1 Northwestern University, Chicago, Illinois, United States; 2 University of Alabama at Birmingham, Birmingham, Alabama, United States

## Abstract

The University of Alabama at Birmingham Life-Space Assessment (LSA) is a self-reported measure for assessing community mobility. Restricted mobility is correlated with a number of adverse health outcomes, including mortality, frailty, cognitive decline, and nursing home admissions. Thus, it is important for providers to understand how the LSA score of a patient compares to the general population. To facilitate such comparisons, we developed demographically adjusted norms for the LSA and its correlation with other functional measures. Norms were based on 15,390 participants age 45 and older in the National Institutes of Health-funded REasons for Geographic and Racial Differences in Stroke (REGARDS) study, a national, population-based, longitudinal study investigating the causes of excess stroke mortality among African Americans and individuals living in the Southeastern US stroke belt region. LSA scores declined from a median of 100 in the 45-54 age range to a median of 59.7 in the 85 and older age range, with higher median scores in males. LSA scores showed modest but significant positive correlations with SF-12 Physical Component and Mental Component, Center for Epidemiologic Studies Depression Scale, and Six Item Screener cognitive scores, as well as modest but significant negative correlations with AD8 Dementia Screening, Katz Activities of Daily Living, and Timed Walk scores. The LSA is a brief, easily administered measure that offers a valid method of assessing community mobility in the older adult population.

